# The combination of neurotropic B vitamins (B1, B6, and B12) is superior to individual B vitamins in promoting neurite growth in vitro

**DOI:** 10.1007/s11626-025-01024-3

**Published:** 2025-03-05

**Authors:** Melissa L. D. Rayner, Arnaud J. Ruiz, Christian Viel

**Affiliations:** 1 UCL Centre for Nerve Engineering, University College London School of Pharmacy, Brunswick Square, London, 29-39 UK; 2 Department of Pharmacology, University College London School of Pharmacy, Brunswick Square, London, 29-39 UK; 3 German Innovation Center, Medical & Technical Affairs, P&G Health Germany GmbH, Sulzbacher Straße 40, 65824 Schwalbach Am Taunus, Germany

Peripheral neuropathy (PN) is the most common disorder of the peripheral nervous system (PNS) in adults. PN symptoms include tingling, numbness, painful sensations, and paraesthesia, negatively impacting patients’ quality of life (Hicks and Selvin 2019). PN can be caused by different underlying etiologies, with diabetes being the most common cause (Callaghan et al. 2015; Nold and Nozaki 2020). Despite the absence of approved drugs to reverse PN, neurotropic B vitamins (B1, B6, and B12) have shown a potential in improving PN conditions (Ghandi *et al*. 2022). For neuronal energy supply, vitamin B1 (thiamine) is crucial in providing adenosine triphosphate (ATP) and/or nicotinamide adenine dinucleotide phosphate (NADPH). Vitamin B6 (pyridoxine) plays a vital role in synthesizing neurotransmitters, enabling signal transmission and modulation in the nervous system. Vitamin B12 (cobalamin) supports nerve regeneration by aiding in myelin synthesis electrically insulating the nerves and ensuring fast signal conduction (Calderon-Ospina and Neva-Mesa 2020). These three vitamins—B1, B6, and B12—act in biochemical synergy and cannot replace each other (Sechi et al. 2016; Geller et al. 2017). This hypothesis is supported by numerous in vitro and animal studies (Jolivalt et al. 2009; Baltrusch 2021), but evidence on how the synergistic mode of action works is still to be explored. We have previously demonstrated that a combination of the neurotropic vitamins B1, B6, and B12 promotes neurite growth in an in vitro neuropathy model of neurodegeneration (Smith et al. 2023). In the present work, we also investigated the effect of the individual B vitamins in comparison to the combination of all three B vitamins with the intention to confirm the synergistic effect of B vitamins on neurite growth of healthy neural cells reported in literature (Calderon-Ospina and Nava-Mesa 2020) (Table 1).

**Table 1.** Absolute values of neurite growth in µm and the respective treatments

Treatment	Mean neurite growth (µm)
Vitamin B free	64.48
Vitamin B1	66.32
Vitamin B6	72.29
Vitamin B12	74.32
Combination (B1, B6, B12)	97.43

Neural cell line NG108-15 (mouse neuroblastoma × rat glioma hybrid neural cell line; Merck Life Science UK Ltd, Feltham, UK, cat no: 88112302-1VL) was cultured in Dulbecco’s modified Eagle medium (DMEM, F12; Thermo Fisher Scientific, Waltham, MA) supplemented with 10% v/v fetal bovine serum (FBS) and 100 U/mL of penicillin and 100 μg/mL of streptomycin in standard cell culture T75 flasks. The cultures were maintained at sub-confluency at 37 °C with 5% CO_2_ and passaged when needed. NG108-15 cells were commercially sourced which are routinely tested by the company and provided with a certificate of analysis. The medium lacking vitamin B1, vitamin B6, and vitamin B12 was prepared (all other vitamins remained): for 500 mL of medium 2250 mg D-glucose, 52 mg L-tyrosine disodium salt dihydrate, 21 mg L-serine, 15 mg glycine, 3.6 mg myo-inositol, 2 mg calcium-d-pantothenate, 2 mg choline chloride, 2 mg folic acid, and 0.05 mg iron(III) nitrate nonahydrate were added to 465 mL Earle’s Balanced Salt Solution (EBSS) (Merck, Rathway, NJ) containing 20 mL minimum essential medium Eagle (MEM) (Merck) (10 ×), 10 mL L-glutamine (200 mM) (Thermo Scientific), and 100 U/mL penicillin and 100 µg/mL streptomycin. B1 (thiamine hydrochloride (Merck)), B6 (pyridoxal hydrochloride (Merck)), and B12 (cyanocobalamin (Merck)) vitamins were added individually or in combination to this medium to obtain concentrations of 40 μM vitamin B1, 20 μM vitamin B6, and 0.4 μM vitamin B12. A concentrated stock solution of the vitamins was added directly to the media in the appropriate volume to provide the required drug concentration. Doses of 40 μM vitamin B1, 20 μM vitamin B6, and 0.4 μM vitamin B12 were used as they had previously shown to be optimal in vitro on healthy mouse dorsal root ganglion (DRG) neurons [54]. NG108-15 cells were seeded at a density of 5000 cells/well onto a 96-well plate in vitamin B-free medium. The plate was loaded into an IncucyteÒ S3 Live-Cell Analysis system (Sartorius, Göttingen, Germany) and analyzed every 1–4 h to monitor neurite growth using phase-contrast imaging for 24 h. The cell cultures were subjected to treatments of 40 μM vitamin B1, 20 μM vitamin B6, and 0.4 μM vitamin B12 in isolation or a combination of all three for 24 h by adding the treatment directly to the media in the appropriate volume to provide the required concentration. The ratio of the combined B vitamins was maintained at 100:50:1. Following overnight fixation with 4% paraformaldehyde (PFA), monolayer cells were washed three times with phosphate buffered saline (PBS) (Sigma-Aldrich, St. Louis, MO) and permeabilized using 0.5% Triton-X-100 (Sigma-Aldrich) in PBS. Washes were repeated before blocking using goat serum (1:20 in PBS; Vector Laboratories, Newark, CA). Gels were washed before the addition of primary antibody of interest (mouse anti-βIII-tubulin 1:400) (Sigma-Aldrich) diluted in PBS overnight at 4 °C. Washes were repeated before adding the corresponding secondary antibody (DyLight anti-mouse IgG 488 1:300 (Vector Laboratories), diluted in PBS). The cells were washed thrice with PBS, and the sample was stored in PBS at 4 °C before viewing. Omission of primary or secondary antibody was routinely used as a control. Micrographs of monolayer cultures were captured using the IncucyteÒ S3 at a × 20 magnification All neurites in the determined fields were manually measured using ImageJ (Schindelin et al. 2012). Normality tests were conducted on all data to determine appropriate statistical tests, and one-way analysis of variance (ANOVA) followed by a post hoc test was performed. *P < 0.05, **P < 0.01, ***P < 0.001, and ****P < 0.0001 were considered to be significant. Data are expressed as means ± SEM when described in the text.

Monolayer NG108-15 cultures were used to analyze the effect of vitamins B1, B6, and B12 in isolation or in combination on neurite growth. Following 24-h treatment, the combination of vitamins B1, B6, and B12 had a significantly greater effect on neurite outgrowth than the individual B vitamins or no treatment (Fig. 1). As shown in Fig. 1, neurite growth was increased highly significantly by 51.10% with the B1, B6, and B12 combination compared to the vitamin B-free control (*P* < 0.001). Treatment with each of the single vitamins, either B1, B6, or B12, did not have a significant influence (*P* > 0.05) on neurite growth compared to the vitamin B-free control with increases of 2.85%, 12.11%, and 15.25% respectively. The effect on neurite growth was 3.35-fold higher with the combination of B1, B6, and B12 compared to the effect of vitamin B12. In comparison, the combination of B1, B6, and B12 elevated neurite growth 4.22-fold more than treatment with vitamin B6 and 17.92-fold more than treatment with vitamin B1. The fluorescence micrographs of immunostained monolayer culture of NG108-15 cells (Fig. 2) visually indicated superiority in regard to neurite extension by the combination of vitamins B1, B6, and B12 compared to the treatment with each of the individual vitamin B1, B6, or B12. The quantitative analysis of the micrographs is reflected in Fig. 1. For this analysis, only the neurite length was quantified whereas the number of cell bodies or the cell body area was not considered. Neurite growth is essential for nerve connectivity and the function of the nervous system.

**Figure 1.**
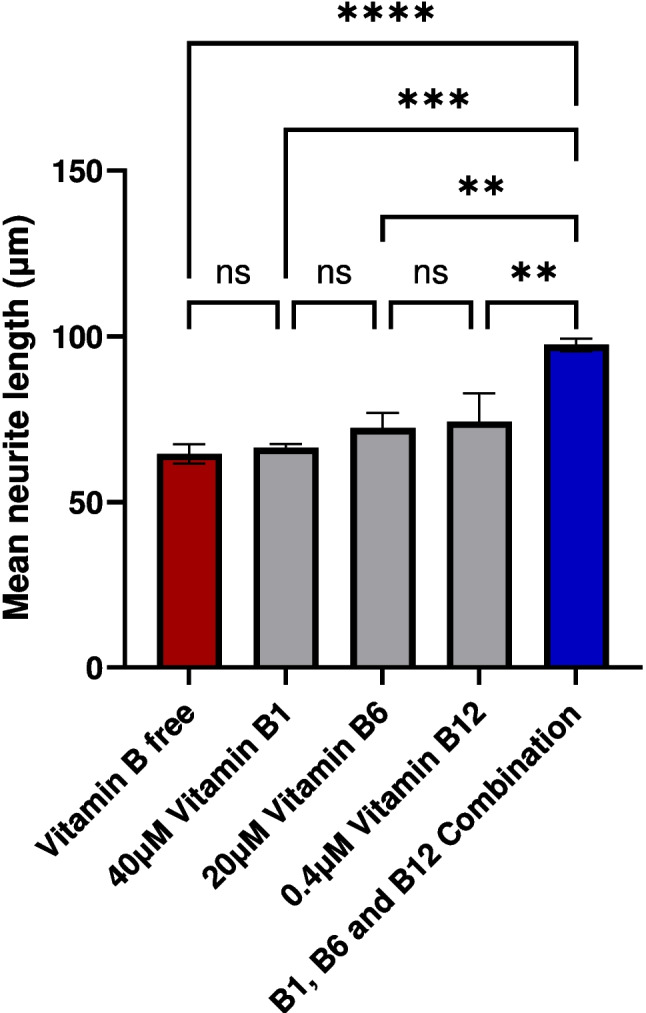
Neurite length was measured at 24 h following treatment in the absence or presence of vitamins B1, B6, and B12 applied as single vitamins or as a combination of all three vitamins. Neurite length was significantly higher when the NG108-15 cells were treated with the combination of vitamins B1, B6, and B12 than with each B vitamin alone or no treatment. *N* = 6–9; mean ± SEM. One-way ANOVA followed by a Tukey post hoc test, **P < 0.01, ***P < 0.001, ****P < 0.0001.

**Figure 2.**
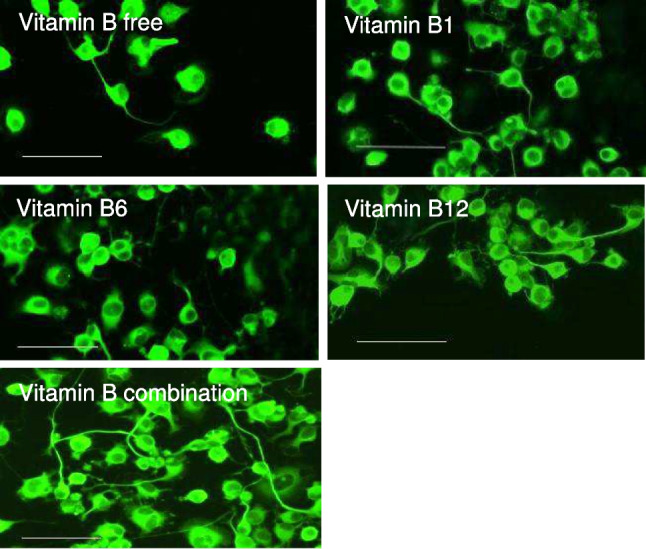
Immunofluorescence images of monolayer NG108-15 cells treated with or without B vitamins. Cells were stained with βIII-Tubulin (*green*). *Scale bar* = 100 μm.

Due to their pivotal role in the nervous system, neurotropic B vitamins are critical for maintaining neuronal viability and contribute essentially to nerve health, function, and growth. They are also critical for network formation which ensures connectivity of nerve cells and with this, normal functioning of the network. Each of the neurotropic B vitamins plays an important role and acts via a unique mode of action, contributing to the neuronal health. Within an in vitro model of NG108-15 cells, this study suggests that all of the three B vitamins act in biochemical synergy to promote neurite outgrowth in healthy neural cells. The significant increase in neurite growth that was only demonstrated in the cells that received the combination of all three B vitamins (B1, B6, and B12) supports the synergy hypothesis suggested by Calderon-Ospina and Nava-Mesa (2020). In fact, the increases in neurite growth observed with either vitamin B1, B6, or B12 do not add up to the effect observed with the combination of B1, B6, and B12. On the contrary, the observed effect with the combination of B1, B6, and B12 clearly exceeds that of the additive effect of each B vitamin.

On a molecular level, these abilities are likely mediated by differentially expressed genes, which regulate levels of proteins and enzymes involved in metabolic processes, transcription, neuron death, and other pathways (Banek et al. 2022). Beyond that, when nerves get damaged, vitamins B1, B6, and B12 play a critical role in supporting nerve regeneration, which would be less successful without these B vitamins as shown previously (Smith et al. 2023).

In summary, our results demonstrate the significant effect and crucial role of the combination of vitamins B1, B6, and B12 on neurite growth of healthy neurons which is significantly superior to each individually administered B vitamin within the NG108-15 cell model. Since each neurotropic B vitamin has a unique mode of action in supporting nervous system health, it makes sense that the combination acts in biochemical synergy. Future experiments will further investigate the superiority of the combination of vitamins B1, B6, and B12 and the underlying mechanism of synergy.

## Data Availability

The data presented in this study are available on request from the corresponding author.
